# Large scale sequence alignment via efficient inference in generative models

**DOI:** 10.1038/s41598-023-34257-x

**Published:** 2023-05-04

**Authors:** Mihir Mongia, Chengze Shen, Arash Gholami Davoodi, Guillaume Marçais, Hosein Mohimani

**Affiliations:** 1 School Computer Science, Carnegie Mellon University, Pittsburgh, USA; 2 Apple. Cupertino, California, USA; 3 University of Illinois, Urbana-Champaign, USA

**Keywords:** DNA sequencing, Computational biology and bioinformatics

## Abstract

Finding alignments between millions of reads and genome sequences is crucial in computational biology. Since the standard alignment algorithm has a large computational cost, heuristics have been developed to speed up this task. Though orders of magnitude faster, these methods lack theoretical guarantees and often have low sensitivity especially when reads have many insertions, deletions, and mismatches relative to the genome. Here we develop a theoretically principled and efficient algorithm that has high sensitivity across a wide range of insertion, deletion, and mutation rates. We frame sequence alignment as an inference problem in a probabilistic model. Given a reference database of reads and a query read, we find the match that maximizes a log-likelihood ratio of a reference read and query read being generated jointly from a probabilistic model versus independent models. The brute force solution to this problem computes joint and independent probabilities between each query and reference pair, and its complexity grows linearly with database size. We introduce a bucketing strategy where reads with higher log-likelihood ratio are mapped to the same bucket with high probability. Experimental results show that our method is more accurate than the state-of-the-art approaches in aligning long-reads from Pacific Bioscience sequencers to genome sequences.

## Introduction

Aligning millions of DNA sequences is significant for identifying functional and evolutionary relationships between organisms, assembling genomes, and analyzing single-nucleotide polymorphisms^1,18^. Many methods have been developed for solving the sequence alignment problem over the past decades^3,4,7,9–12,15^. Currently, the most popular alignment algorithms use the seed-chain alignment procedure. In this procedure a set of subsequences are extracted from the reference genome and indexed. Likewise a set of subsequences are extracted from a batch of query reads. Then each query read is only compared to sections of the reference genome that have subsequences in common. The assumption is that each read can only map to sections of the genome which share exact subsequences with the read. This assumption, however, is violated when error rate is high and thus the most popular alignment algorithms fail to identify a large portion of true alignments. One way to identify more true alignments in the high error rate regime is to relax the requirement that read and genome share exactly matching subsequences to read and genome share similar subsequences (not identical). However, methods for speeding up alignments of reads with high error rates using this relaxation are not available.

**Figure 1:**
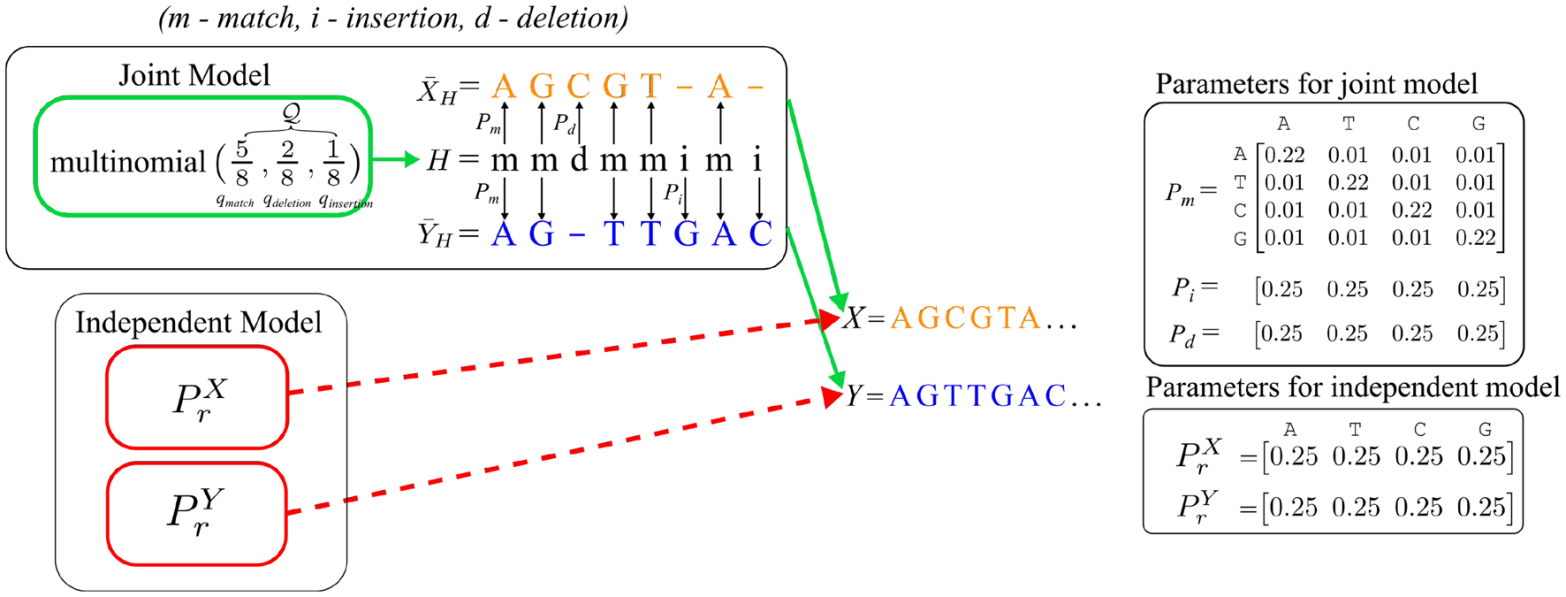
We model sequence alignment as an inference problem in a latent variable model. We assume the true alignments are generated through a joint model, while random pairs are generated through independent models. In the joint model, a latent variable $H = \{i,m,d\}^{S}$ is first sampled from a multinomial distribution. Here, “*m*”, “*i*”, and “*d*” represent match/mismatch, insertion and deletion. Then sequences ${\bar{X}}_{H}$ and ${\bar{Y}}_{H}$ are generated based on sequence *H* and probability matrices $P_{m}$, $P_{i}$, and $P_{d}$. Whenever we have “*m*” at a position in *H*, the corresponding positions in ${\bar{X}}_{H}$ and ${\bar{Y}}_{H}$ are sampled jointly from $P_{m}$. Whenever we have “*d*” at a position in *H*, the corresponding position in ${\bar{X}}_{H}$ is sampled from $P_{d}$, while we have “−” for ${\bar{Y}}_{H}$. Whenever we have “*i*” at a position in *H*, the corresponding position in ${\bar{Y}}_{H}$ is sampled from $P_{i}$, while we have “−” for ${\bar{X}}_{H}$. Then sequences *X* and *Y* are formed by removing “−” from ${\bar{X}}_{H}$ and ${\bar{Y}}_{H}$. For the independent model, *X* and *Y* are independently sampled from $P_{r}^{X}$ and $P_{r}^{Y}$.

**Figure 2:**
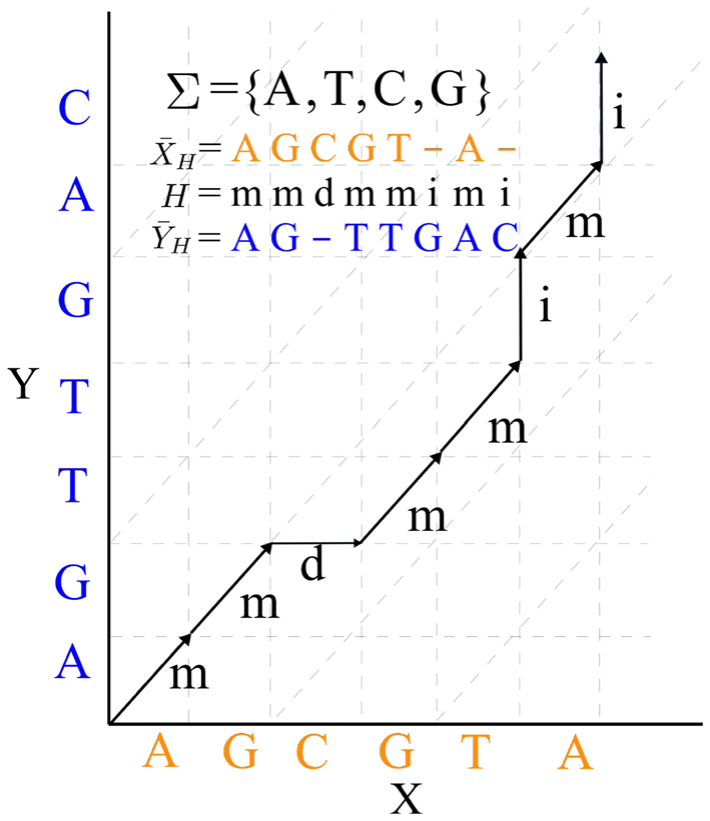
In the joint model, a latent variable $H = \{i,m,d\}^{S}$ is first sampled, and then ${\bar{X}}_{H}$ and ${\bar{Y}}_{H}$ are generated based on *H*. *X* and *Y* are generated by removing “−” from ${\bar{X}}_{H}$ and ${\bar{Y}}_{H}$. Here, “*m*”, “*i*” and “*d*” stand for match/mismatch, insertion and deletion.

Sequence alignment has been studied from a statistical inference perspective^8,9^. It has been shown that optimal inference is equivalent to the dynamic programming solution to the sequence alignment problem. In order to overcome the low true positive rate of existing sequence alignment methods when the error rate is high, we first model sequence alignment as an inference problem in a latent variable generative model (Figs.  1 and  2) and then develop “asymetrical” hashing techniques for fast inference in this model. These asymetrical hashes can hash two reads to the same bucket or value whenever the two reads contain a pair of k-mers that are similar (but not necessarily identical). The set of non-matching pairs used for asymetrical hashing is optimally chosen with respect to the latent variable generative model.

In our model, we assume that pairs of sequences with high alignment scores are generated jointly from a generative model, and pairs of sequences with low alignment scores are generated independently of each other. Here we denote the probability that a pair of sequences *X* and *Y* are generated under the joint model as ${\mathbb {P}}(X,Y)$. The probability that *X* and *Y* are generated independently of each other is thus ${\mathbb {P}}^{x}(X){\mathbb {P}}^{y}(Y)$ where ${\mathbb {P}}^{x}$ denotes the marginal of ${\mathbb {P}}$ along the *x* variable and ${\mathbb {P}}^{y}$ denotes the marginal of ${\mathbb {P}}$ along the *y* variable.

Given this model, a natural algorithm to find aligned sequences is the following: for each pair of sequences *X* and *Y*, compute $C_{X,Y} = \frac{{\mathbb {P}}(X,Y)}{{\mathbb {P}}^{x}(X){\mathbb {P}}^{y}(Y)}$. If $C_{X,Y}$ is high, then *X* and *Y* are more likely to be generated from the joint model, rather than the independent model. Therefore, $C_{X,Y}$ can be used as an alignment score between *X* and *Y*.

A naive implementation of this natural algorithm suffers from the fact that $C_{X,Y}$ needs to be computed for each pair of sequences *X* and *Y* in the database. To overcome this challenge, we further propose a bucketing method inspired by locality sensitive hashing. In this strategy, we put each sequence *X* into several buckets (likewise for *Y*). For each *X*, we compute $C_{X,Y}$ only for the sequences *Y* that are in the same buckets as *X*. This strategy significantly reduces the number of sequence pairs for which $C_{X,Y}$ is computed, without missing true positive pairs.

In this paper, we introduce Distribution Sensitive Bucketing (DSB), a novel technique for efficient and accurate alignment of large read datasets against genomes. To do this, we first formulated the sequence alignment problem as an inference in two types of latent variable models, Hidden Markov Models (HMMs) and pair Hidden Markov Models (pair-HMMS). For both probabilistic models, we developed an efficient inference algorithm and derived its complexity. We further designed a family of asymmetric bucketing functions that minimize the complexity. Several alignment software packages have been developed using HMM like probabilistic models^1–3^. Algorithms based on HMM like probabilistic models are either slow or require heuristics^1,3^ with no theoretical bound on performance. The area of asymetric bucketing is relatively underexplored^4^. In order to detect homolog sequences, Mak et al.^4^ introduced a method that would put pairs of sequences in the same bucket if they shared the same subsequence plus or minus a few deletions and insertions. However, Mak et al. does not consider that computational complexity may increase due introducing this procedure and do not provide a methodology that adjusts the subsequence length and number of indels considered to various indel probabilities.

DSB is an algorithm that given indel probabilities will generate a bucketing data structure for sequence alignment that aims to keep sensitivity high while keeping computational complexity of sequence alignment low. Like Mak et al., DSB will compare reads that share similar subsequences, not just identical ones. Our results show that the DSB algorithm is at least as sensitive as competing methods in aligning reads at low error rates, and outperforms all methods when the error rates are high. When aligning reads to distant homologs, the DSB algorithm is 10 percent more sensitivity than the closest competitor.

For the sake of presentation clarity, we provide bodies of algorithms in Supplementary Materials and their summaries in the main text. Additional figures and tables are also provided in Supplementary Materials.

## Results

We benchmarked DSB-SA against popular alignment methods in several scenarios. The scenarios include aligning simulated reads against a reference genome, aligning experimental reads against corresponding genomes, and aligning *E. coli* reads against reference genomes of distance homologs. In each scenario both Sensitivity and False Positives are reported. Sensitivity is measured as the proportion of aligned regions (read to genome) in the reference that are reported by the corresponding method. A sensitivity of 1 means that every region in the reference is recovered while 0 means no regions in the genome are recovered.

### Brief overview of distribution sensitive bucketing

Distribution Sensitive Bucketing (DSB) consists of the following steps (see details in the Method Section). First, a directed graph is constructed, where each sink node represents a bucket. Then, starting from the source node, query and reference reads are mapped to one or several buckets through the decision graph (at each node in the graph, the decision graph might route a read to more than one direction). Finally, all the buckets are explored, and pairs of query and reference reads in each bucket are reported as matches. In the following, DSB-HMM refers to DSB algorithm in case of HMM model (Algorithms 1 and 2), while DSB-SA refers to DSB algorithm in case of string alignment model (Algorithms 3 and 5).

We benchmarked DSB-SA against MHAP^5^, Minimap2^6^, DALIGNER^7^, BlasR^8^, MMSeqs2^9^, GraphMap^10^, CD-HIT^11^, and Winnowmap^12^. MHAP is the state of the art algorithm for aligning reads with high insertion, deletion, and mismatch rates, that is based on compressing sequences to their representative fingerprints and detecting overlaps by estimating Jaccard’s similarity using min-wise hashing^13^. Minimap2 is a general-purpose alignment program for mapping DNA reads to reads/large reference databases, that is based on collecting and indexing minimizers in a hash table^6^. DALIGNER is based on an efficient and highly sensitive filter that predicts points between pairs of reads that are likely to have a significant local alignment passing through them^7^. BlasR first finds clusters of short exact matches between the read and the genome using a suffix array, and then perform a more detailed alignment of the regions where reads are matched^8^. MMSeqs2 is a sensitive and fast alignment program that utilizes k-mer matching and ungapped alignment to speed up mapping without losing sensitivity^9^. GraphMap is a fast and sensitive reads mapping program which is designed to analyze sequence data with high error such as nanopore sequencing data^10^. CD-HIT is a clustering algorithm that group reads together based on their pair-wise similarity^11^. Winnowmap is a direct descendent of Minimap2, and it uses weighted frequently occurring k-mers to reduce excessive false positives^12^.

### Benchmarking DSB-HMM in matching simulated data

In this experiment, we compared the performances of DSB-HMM (Algorithms 1 and 2) against the brute force search (Algorithm 4). Data is simulated from a HMM with hidden state $h_{t}\in \{0,1\}$ for $0\le t \le T$ and the following parameters:

1 $$\begin{aligned} P_{trans}&= \left[ \begin{matrix} 1-\delta &{} \delta \\ \delta &{} 1-\delta \end{matrix}\right] \end{aligned}$$

2 $$\begin{aligned} P_{emit}(x_{t},y_{t}\mid h_{t}=0)&= \left[ \begin{matrix} 0.5-\epsilon &{} \epsilon \\ \epsilon &{} 0.5-\epsilon \end{matrix}\right] \\ P_{emit}(x_{t},y_{t}\mid h_{t}=1)&= \left[ \begin{matrix} \epsilon &{} 0.5-\epsilon \\ 0.5-\epsilon &{} \epsilon \end{matrix}\right] \nonumber \end{aligned}$$

where $P_{trans}$ represents the transition matrix of the hidden states, i.e. $P_{trans}(i,j) = P(h_{t+1} = j \mid h_{t} = i)$ and $P_{emit}$ represents the emission probabilities, i.e. $P_{emit}(i,j \mid h)=P(x_{t} = i, y_{t} = j \mid h_t=h)$. Here, $\epsilon$ is the error rate, and $\delta$ is the probability of transition to an alternative state. In hidden state 0, the HMM mostly generates matching nucleotides whereas in hidden state 1 the HMM generates mostly mismatching nucleotides.

We first simulate the hidden states using the transition probabilities, and then simulate data using the emission probabilities for various values of $\epsilon$ and $\delta$. We run for 1000, 2000, 5000, 10 000, 50 000, 100 000, and here $T=2000$. The brute force algorithm has a quadratic growth in complexity with respect to the number of sequences *N*, while our method has a sub-quadratic complexity (Fig. S1).

### Benchmarking DSB-SA in aligning simulated reads against genomes

**Figure 3:**
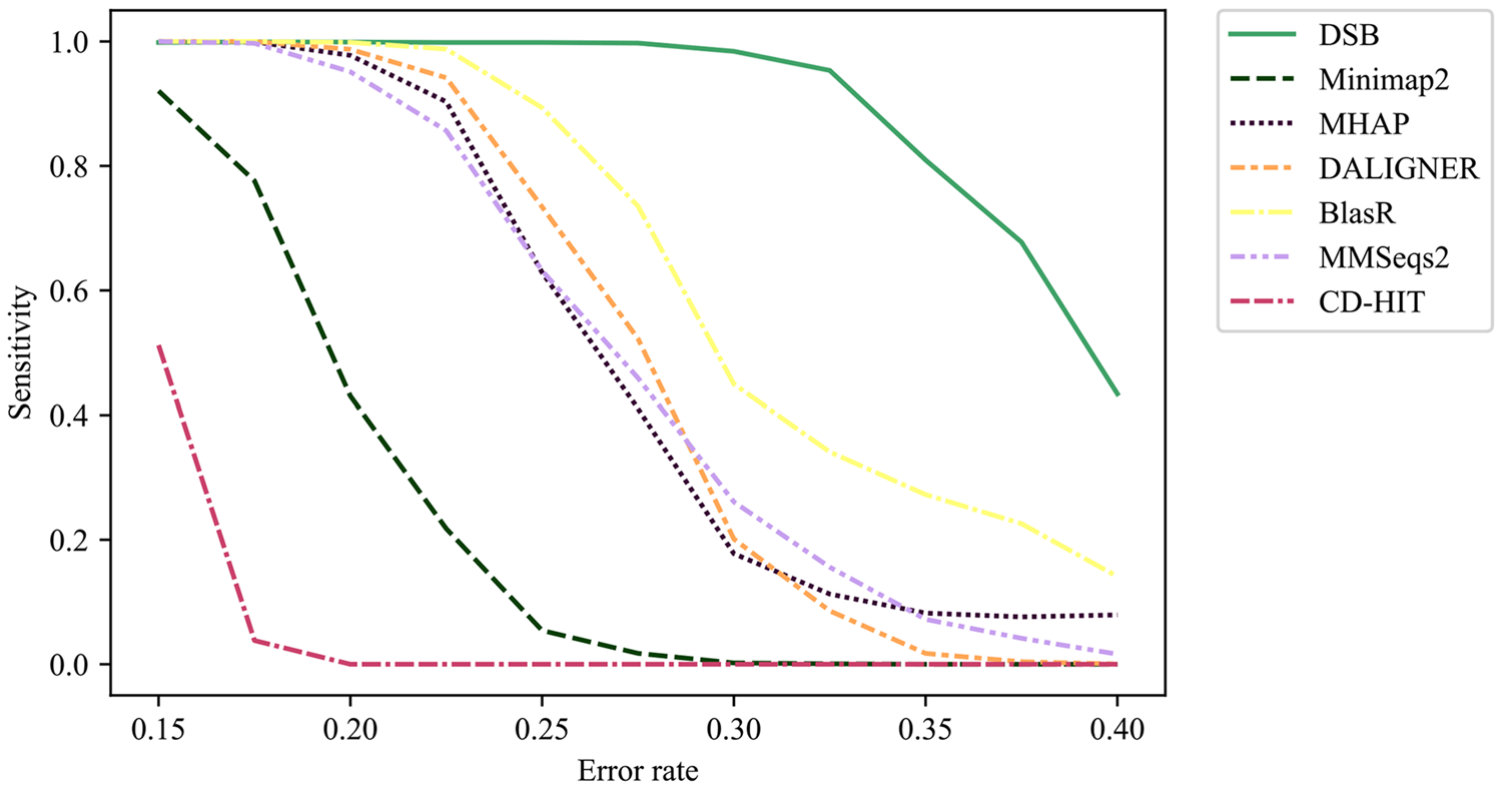
Benchmarking different methods on mapping simulated reads against the genome. We use PBSIM to simulate PacBio reads from the *E. coli* reference genome. Indel and substitution errors are introduced, and the error rate on reads is the sum of these errors. In this experiment, we simulated the reads with an average length of 700 bps and average read errors from 0.25 to 0.45. Sensitivity versus error rate for DSB-SA, Minimap2, Winnowmap, DALIGNER, BlasR, MMSeqs2, and GraphMap are shown. Details of the PBSIM simulation are provided in the Supplementary Note 4.

We benchmarked the different methods in mapping $N=5000$ PacBio simulated reads against the *E. coli* reference genome. We used recommended settings for all methods. The reads are simulated using PBSIM^14^ and the *E. coli* reference genome, with a mean length of 700 bps. Figure 3 shows the sensitivity, the percentage of reads mapped correctly to the genome.

**Figure 4:**
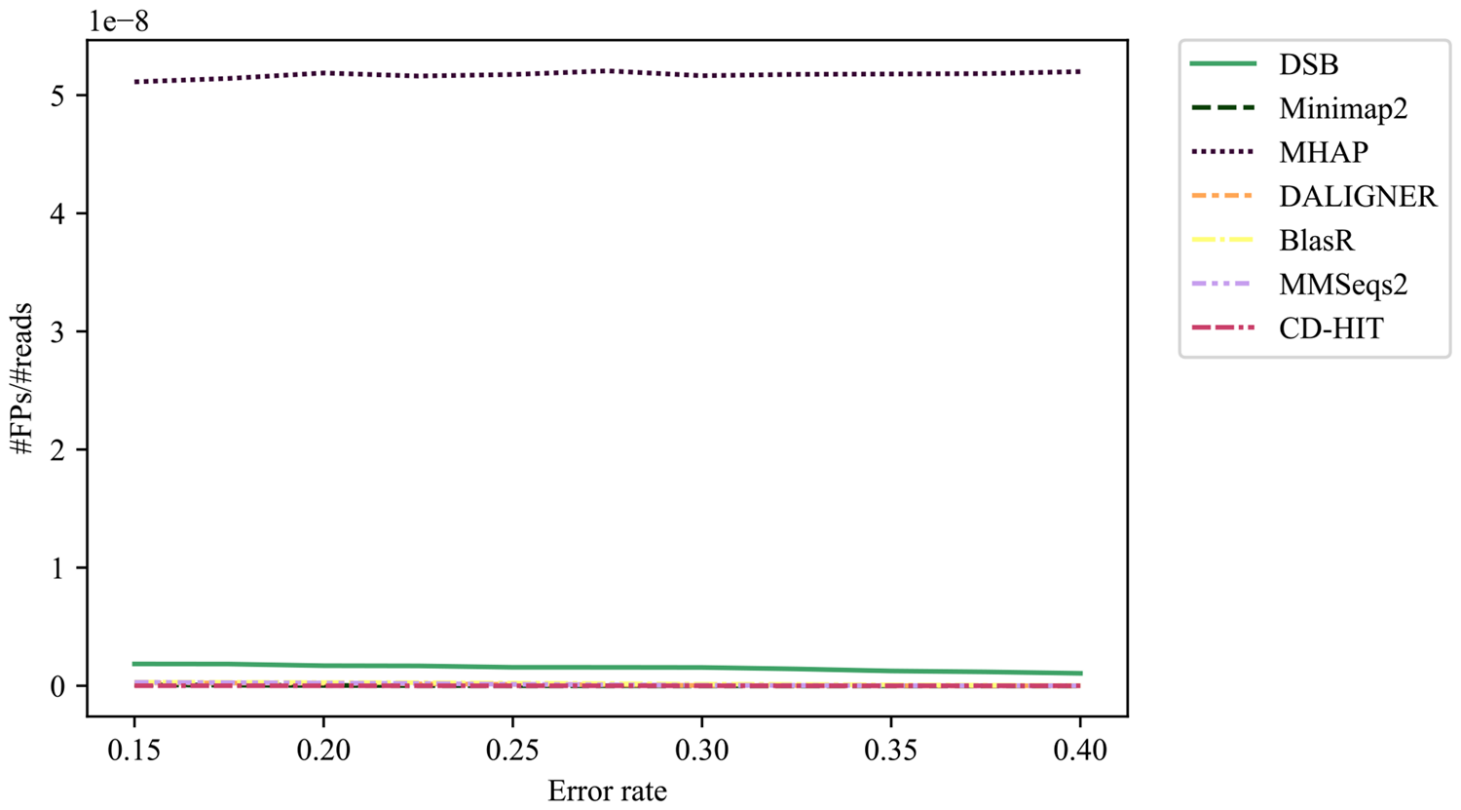
Benchmarking the false positive rate of different methods on mapping simulated reads against the genome. We use PBSIM to simulate PacBio reads from the *E. coli* reference genome. Indel and substitution errors are introduced, and the error rate on reads is the sum of these errors. In this experiment, we simulated the reads with an average length of 700 bps and average read errors from 0.15 to 0.40. False positive rate of all methods is nearly zero.

We observe that DSB-SA maintains high sensitivity as the error rate increases. BlasR has a lower sensitivity compared to DSB-SA, and its performance decreases substantially for higher error rates. DALIGNER, Minimap2, Winnowmap and MMSeqs2 show similar low sensitivity across different error rates. The false positive rate for all methods is nearly zero (Fig. 4).

### Benchmarking DSB-SA in aligning pacbio reads to distant homologs

**Figure 5:**
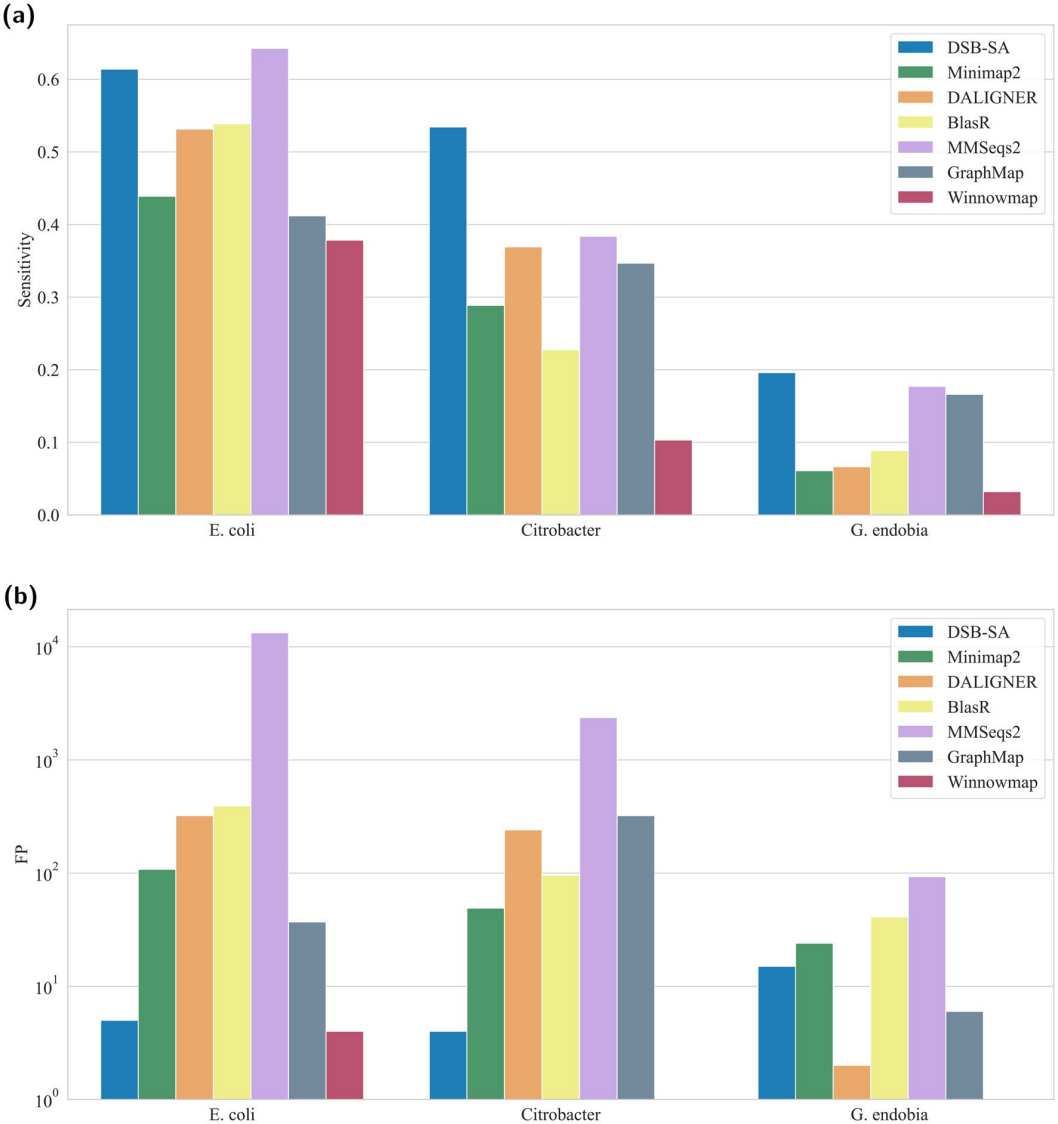
Comparison of sequence alignment methods in (**a**) sensitivity and (**b**) false positives. Here, Pacbio reads from *E. coli* are searched against the genome of *Citrobacter* and *G. endobia* to assess the power of various methods in identifying distant homologs.

In this experiment, we compared the performances of DSB-SA with various methods on mapping PacBio reads of the *E. coli* genome to two genomes of related species. The ground truth is inferred by aligning the reads against the genomes with LALIGN^15^. LALIGN is a brute force method that finds all local alignments between given queries and targets. We use default settings for DALIGNER, BlasR, MMSeqs2, GraphMap and Winnowmap, and PacBio preset for Minimap2. Figure 5 illustrates the sensitivity of the examined methods.

**Table 1:** Runtime/memory analysis of various methods for mapping PacBio long reads of *E. coli* to the *E. coli* genome.

*E. coli*		
	runtime (s)	memory (MB)
LALIGN	1587806.20	-
DSB-SA	174.22	2022.54
Minimap2	1.79	71.14
DALIGNER	11.92	761.04
BlasR	482.95	104.89
MMSeqs2	64.80	8314.44
GraphMap	102.53	369.94
Winnowmap	1.41	60.05

The runtime is recorded in seconds (s), and the memory usage is recorded in megabytes (MB).

With the exception of MMSeq2, DSB-SA is significantly more sensitive than the other methods (Fig. 5a). While DSB-SA is nearly as sensitive as MMSeq2, it produced three orders of magnitude fewer false positives (Fig. 5b). Runtimes for all the methods are shown in Table 1. False negatives and true positive rates for all methods are shown in Supplementary Tables S1, S2, and S3.

## Benchmarking DSB-SA in aligning PacBio reads to corresponding genomes

**Figure 6:**
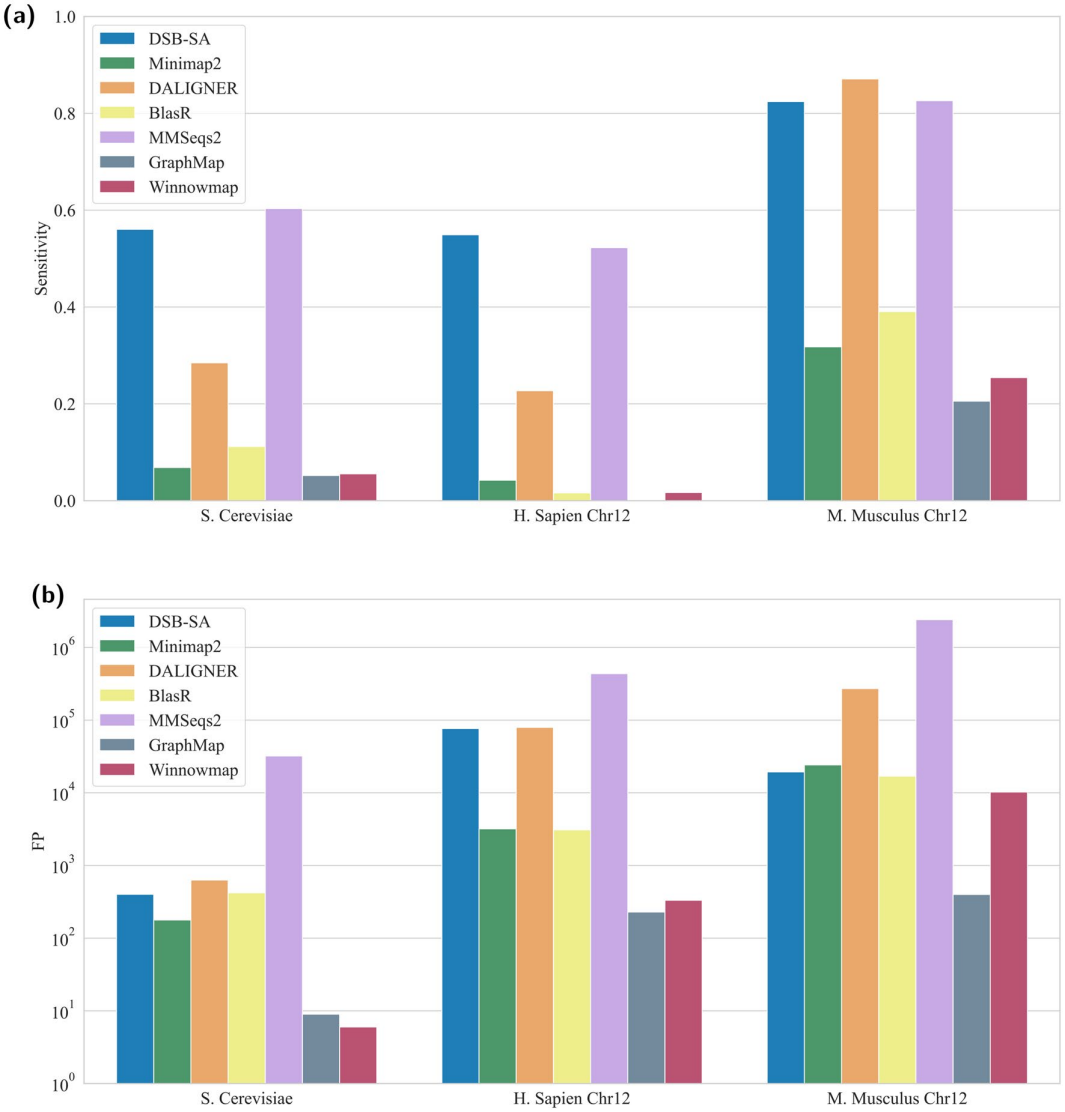
Comparison of (**a**) sensitivity and (**b**) false positives of various sequence alignment methods. Here, Pacbio reads from *S. Cerevisiae*, *H. Sapien*, and *M. Musculus* are searched against their respective genomes.

We benchmarked DSB-SA against other methods in mapping PacBio reads of *S. Cerevisiae*, chromosome 12 of *H. Sapiens*, and chromosome 12 of *M. Musculus* to their respective genomes (Fig. 6). DSB-SA is significantly better than all the other methods with exception of MMSeq2 and DALIGNER. DSB-SA is comparable (or better) than MMSeq2 and DALIGNER, and produces significantly fewer false positives. False negatives and true positive rates for all methods are shown in Supplementary Tables S4, S5, and S6.

## Discussion

In recent years, various efficient methods have been introduced for aligning large number of reads against reads/genomes. However, majority of these techniques are limited to the cases where reads are mapped to close homologs (low mutations, insertions and deletions) and they do not generalize to alignment of reads to distant homologs. Furthermore, most of the methods that are used in practice use heuristics to speed up alignment.

Many approaches also use decision trees to solve the string alignment problem. The main contribution of the presented work is in modeling the string alignment problem as a statistical inference problem and applying a general hashing strategy for efficient inference. Since we use pair-HMMs to model the joint distribution of aligned reads, the final hidden state of a pair of aligned reads can be a deletion, insertion, or match/mismatch. Thus, there are three pairs of subsequences that are predecessors of a sequence pair (note that in traditional HMM, there is only a single predecessor). Thus the hashes for pair-HMMs are *decision graphs* (Fig. S4) rather than decision trees.

The sequence alignment problem has been modeled as a statistical inference problem, and dynamic programming solutions have been proposed to achieve optimal inference. Since these statistical models can be parameterized by different insertion, deletion, and mutation rates, the dynamic programming solutions are capable of discovering alignments between distant homologs. Still dynamic programming is very slow and runtime grows linearly with the size of reference. Inspired by locality sensitive hashing, in this paper we introduced the distribution sensitive bucketing paradigm for efficient subquadratic inference in latent variable models. In this paradigm, given a joint and an independent model for true and random pair of sequences, the sequences are mapped to buckets in a way that pairs of sequences from the joint model are more likely to fall into the same bucket than random ones. By focusing on pairs that fall into the same bucket, Distribution Sensitive Bucketing (DSB) can not only find read/genome pairs over a wide range of insertion, deletion, and mutation rates, but also avoids many unnecessary computations.

Brute force techniques (e.g. LALIGN) are incapable of handling large scale alignment tasks. On the other hand, faster methods (e.g. Minimap2 and DALIGNER) can not handle larger mismatch, insertion and deletion rates. DSB is capable of recovering a significant portion of alignments with a reasonable computation time. We do not advise using DSB in cases where error rates are small (e.g. Illumina reads). In these scenarios, our benchmark shows that Minimap2 outperforms other methods while maintaining high accuracy. In case of erroneous reads or distant homologs, DSB algorithm achieves high sensitivity, while being orders of magnitude faster than the brute force alternatives.

An alternative strategy to the MinHash approach used in MHAP is densified MinHash^16^. However, our results show that the main disadvantage of MinHash based methods is in their low sensitivity and high false positive rate. Switching to densified MinHash can make this problem more severe, since it has been reported that densified MinHash slightly improves the speed of MinHash while sacrificing sensitivity^17^.

Currently, DSB is based on a simplistic assumption that insertions and deletions occur independently. The current approach paves the path toward more sophisticated models, such as affine gap penalties and non-uniform distribution of nucleotides.

## Methods

### Inference problem for hidden Markov models

Consider an HMM ${\mathbb {P}}$ with a finite alphabet ${\mathcal {H}}$ of hidden states and an alphabet ${\mathcal {A}} \times {\mathcal {B}}$ of observations where ${\mathcal {A}}$ and ${\mathcal {B}}$ are finite discrete alphabets. For any pair of sequences $X = (x_{1},x_{2}, \ldots , x_{T}) \in {\mathcal {A}}^{T}$, $Y = (y_{1},y_{2}, \ldots , y_{T})\in {\mathcal {B}}^{T}$ where $T \in {\mathbb {N}}$, let

3 $$\begin{aligned} {\mathbb {P}}(H)= & {} P_{init}(h_{1})\prod _{t=1}^{T-1}P_{trans}(h_{t+1} \mid h_{t} ) \end{aligned}$$

4 $$\begin{aligned} {\mathbb {P}}(X,Y \mid H)= & {} \prod \limits _{t=1}^T P_{emit}(x_{t}, y_{t} \mid h_{t})\end{aligned}$$

5 $$\begin{aligned} {\mathbb {P}}(X,Y)= & {} \sum _{H \in {\mathcal {H}}^{T}}{\mathbb {P}}(X,Y \mid H){\mathbb {P}}(H) \end{aligned}$$

where $H = (h_{1},h_{2}, \ldots , h_{T}) \in {\mathcal {H}}^{T}$ denotes hidden states, $P_{emit}:{\mathcal {A}}\times {\mathcal {B}} \times {\mathcal {H}} \rightarrow [0,1]$ denotes the emission probabilities, $P_{trans}:{\mathcal {H}}\times {\mathcal {H}}\rightarrow [0,1]$ denotes the transition probabilities, and $P_{init}:{\mathcal {H}} \rightarrow [0,1]$ denotes the initial probability of the hidden state $h_{1}$. Here all multi-dimensional probabilities are shown with ${\mathbb {P}}$, while scalar probabilities are shown with *P*. In the context of sequence alignment, one can treat *Y* as the query sequence and *X* as a sequence in the database. The inference problem for latent variable models is defined as follows: Given a latent variable model ${\mathbb {P}}(X,Y)$, a set of sequences ${\mathcal {X}}=\{X^{1},\ldots ,X^{N}\}\subseteq {\mathcal {A}}^T$, and a query sequence $Y\in {\mathcal {B}}^T$, find $X^{*}$ satisifying:

6 $$\begin{aligned} X^{*} = \mathop {{\mathrm{arg\,max}}}\limits \limits _{X\in {\mathcal {X}}} {\mathbb {P}}(Y \mid X) \end{aligned}$$

where ${\mathbb {P}}(X)$ is the product of background probabilities of each base and insertions/deletions. In the case where ${\mathbb {P}}(X,Y)$ is a HMM defined in (3)-(5), the naive method to solve this optimization problem requires computing ${\mathbb {P}}(Y\mid X^{i})$ for each $X^{i}\in {\mathcal {X}}$ using the classic forward algorithm^18^, and then finding $X^{i}$ that maximizes ${\mathbb {P}}(Y\mid X)$ (see Supplementary Algorithm 4). However, since the complexity of the forward algorithm is $O(T|{\mathcal {H}}|^{2})$, the complexity of this naive method is $O(NT|{\mathcal {H}}|^2)$ (computing *P*(*Y*|*X*) for all $X \in {\mathcal {X}}$). This solution thus cannot scale to cases where the number of sequences *N* is large. Also note that the forward algorithm is not applicable to the string alignment problem with insertions and deletions.

### Database search by distribution sensitive bucketing for HMMs

Here we introduce decision trees with bucket data structures, and show how they can be used for mapping database sequences $X \in {\mathcal {X}}$ and query sequences $Y \in {\mathcal {Y}}$ to buckets in a way that for pairs (*X*, *Y*) with higher ${\mathbb {P}}(Y \mid X)$, the chance of mapping to the same bucket is higher. Intuitively, each node in the tree contains two subsequences $S_x$ and $S_y$, and $X\in {\mathcal {X}}$ (resp. $Y\in {\mathcal {Y}}$) falls in a bucket node in the tree if $S_x$ (resp. $S_y$) is its prefix. We then use this data structure to design an algorithm that solves the inference problem in the case of HMMs.

**Figure 7:**
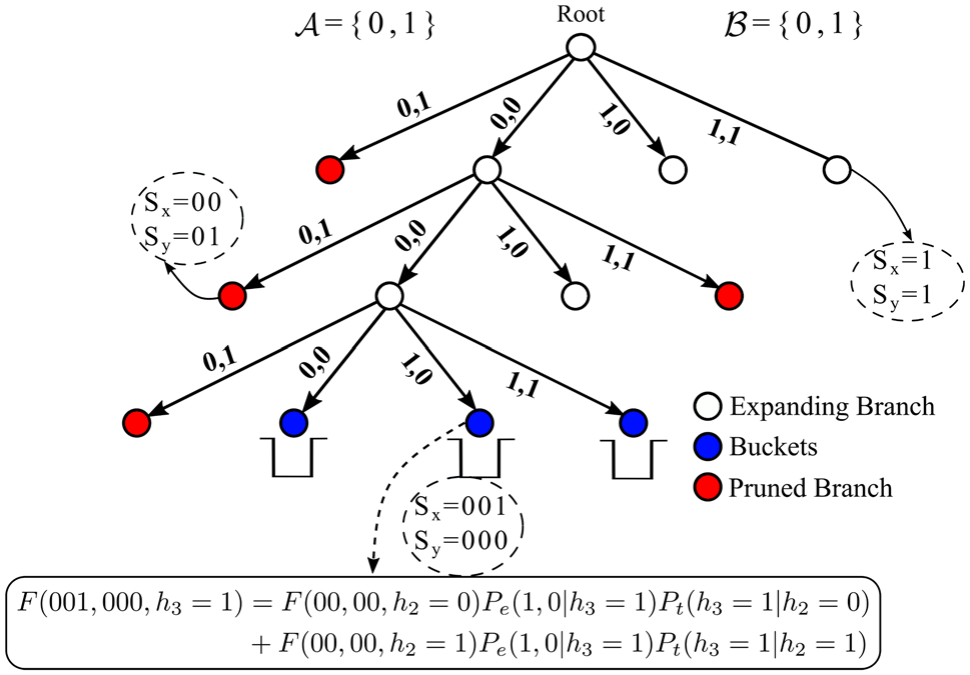
Construction of the HMM decision tree. Examples of accepted, pruned, and branched nodes are shown. Refer to Algorithm 3 for further details of the tree construction.

The data structure consists of a decision tree *G* and a set of distinguished leaf nodes of *G* called “buckets”. Decision tree *G* is a directed tree with a set of nodes *V*. Each node $v \in V$ is associated with two sequences $v.S_{x} \in \cup _{t=1}^{T} {\mathcal {A}}^{t}$ and $v.S_{y} \in \cup _{t=1}^{T}{\mathcal {B}}^{t}$ (called x-sequence and y-sequence of *v*). For each $v \in V$, $v.S_{x}$ and $v.S_{y}$ have the same size, and this size is equal to their depth in the tree. For the root node, $root \in V$, $root.S_{x} = root.S_{y} = \emptyset$, where $\emptyset$ is the empty string. Each node $v \in V$ is either a leaf node, or has exactly $|{\mathcal {A}}| \times |{\mathcal {B}}|$ children. For each child *w* of *v* corresponding to $a \in {\mathcal {A}}, b \in {\mathcal {B}}$, its x-sequence and y-sequence are defined as follows:

7 $$\begin{aligned} w.S_{x} = v.S_{x}+a \quad w.S_{y} = v.S_{y}+ b \end{aligned}$$

where plus sign stands for string concatenation. In other words, $w.S_{x}$ and $w.S_{y}$ are formed by attaching *a* and *b* to the end of $v.S_{x}$ and $v.S_{y}$ strings, respectively. The edge from *v* to *w* is indexed by (*a*, *b*). Furthermore the set of *D* buckets $v\in V_{buckets} = \{v_{1}, \ldots , v_{D} \}$ is a subset of the leaf nodes of the tree, where *D* is referred to as the number of buckets. Later we will discuss how to set *D* and $V_{buckets}$ in order to optimize accuracy and efficiency. Figure 7 shows the schematic of this data-structure for ${\mathcal {A}} = {\mathcal {B}} = \{0,1\}$. Note that the proposed tree differs from suffix/prefix trees in that they are constructed from two distinct databases of strings, and each node in the tree corresponds to a pair of substrings.

Given the decision tree *G* and a set of buckets $V_{buckets}$, we define a natural bucketing strategy for database and query sequences in ${\mathcal {A}}^{T}$ and ${\mathcal {B}}^{T}$. Let’s define:

8 $$\begin{aligned} \text{ Prefix}^{x}(v,X,j)= & {} {\left\{ \begin{array}{ll} 1, &{} \quad v.S_{x} \text{ is } \text{ a } \text{ prefix } \text{ of } j'\text{ th } \text{ suffix } \text{ of } X\\ 0, &{} \quad \text {otherwise} \end{array}\right. }\end{aligned}$$

9 $$\begin{aligned} \text{ Prefix}^{y}(v,Y,j)= & {} {\left\{ \begin{array}{ll} 1, &{} \quad v.S_{y} \text{ is } \text{ a } \text{ prefix } \text{ of } j'\text{ th } \text{ suffix } \text{ of } Y\\ 0, &{} \quad \text {otherwise} \end{array}\right. } \end{aligned}$$

A sequence $X \in {\mathcal {X}}$ maps to a bucket *v* in position $1 \le j \le J$ if and only if $v.S_{x}$ is a subsequence of *X* starting at *j*, i.e. $\mathop {{\textrm{Prefix}}}\limits ^{x}(v,X,j) =1$. A sequence *Y* is mapped similarly. For example if $v.S_{x} = 1011$ and $X = 01011100$, then $\mathop {{\textrm{Prefix}}}\limits ^{x}(v,X,2) = 1$ while $\mathop {{\textrm{Prefix}}}\limits ^{x}(v,X,j) = 0$ for any $j \ne 2$. We will refer to each position *j*, $1 \le j \le J$ as a band. We further refer to the set of bands as ${\mathcal {J}}$ (here ${\mathcal {J}}=\{1,\cdots ,J\}$). Intuitively, only a small ratio of true positives fall into the same bucket in a single band, and therefore *J* bands are needed in order to guarantee a true positive rate nearly one.

Let’s define the bucketing function $h^{x}_{j}(X)$ (resp. $h^{y}_{j}(Y)$) as the set all the buckets in the decision tree that a sequence belongs to from its *j*-th band. That is:

10 $$\begin{aligned} h^{x}_{j}(X)= & {} \{ v \in V_{buckets} \mid \text{ Prefix}^{x}(v,X,j)=1\} \end{aligned}$$

11 $$\begin{aligned} h^{y}_{j}(Y)= & {} \{ v \in V_{buckets} \mid \text{ Prefix}^{y}(v,Y,j)=1\} \end{aligned}$$

Given these buckets we can then solve the inference problem using Algorithm 1. In summary, Algorithm 1 first maps all sequences in ${\mathcal {X}}$ and ${\mathcal {Y}}$ to appropriate buckets, which are obtained by growing and pruning the decision tree *G* (Fig. S3). Then, it examines all pairs in each bucket using forward algorithm to compute ${\mathbb {P}}(X,Y)$, ${\mathbb {P}}^x(X)$ and ${\mathbb {P}}^y(Y)$, and uses the likelihood ratio to determine if the pairs are likely to have an alignment. Since only pairs in each bucket are examined, the complexity is much lower than the brute force strategy from Algorithm 4.
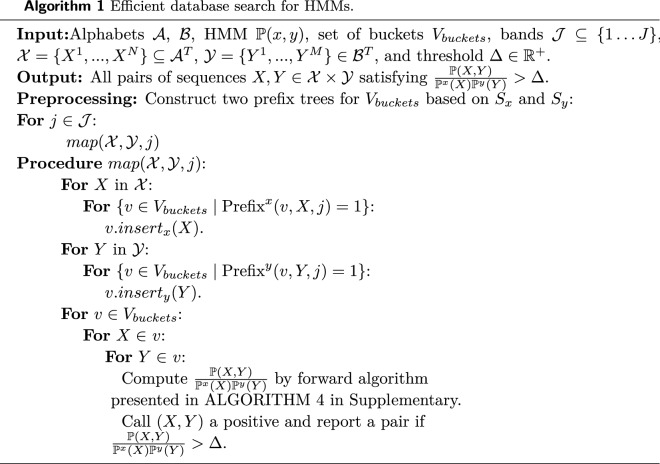


In the special case where for every bucket *v*, $v.S_x = v.S_y$, Algorithm 1 will be restricted to finding exact match substrings based on prefix trees. Enforcing exact match substrings could results in high false negative rates, as distance homolog sequences might not share any substring of certain length. However, by constructing optimal trees and buckets that tolerate errors ($S_x$ not exactly equal to $S_y$), Algorithm 1 can achieve lower false negative rates than methods that enforce exact matches.

Another naive choice of the decision tree and buckets is a complete tree with all leaf nodes selected as buckets. In this case, every pair of sequences will share a bucket, and therefore the complexity of Algorithm 1 would be the same as the brute force algorithm. Now we provide a complexity and true positive rate analysis of Algorithm 1. We further present an algorithm to select a decision tree and a set of buckets, $V_{buckets}$, that minimizes the complexity in Supplementary Sect. 9.6. This algorithm iteratively grows a tree and prunes nodes/buckets that do not have a sufficiently high probability of containing pairs of sequences generated under the joint distribution.

### Complexity and true positive rate analysis

In the Supplementary Materials, we show proofs for the complexity and true positive rate under the HMM model. True positive rate (TPR) of Algorithm 1 is the fraction of (*X*, *Y*) pairs jointly generated under the HMMs that are captured in the same bucket. Using *J* bands we have

12 $$\begin{aligned} \text{ TPR } = 1 - (1 - \alpha )^{J} \ge 1 - e^{-\alpha J} \end{aligned}$$

where $\alpha$ is defined as the true positive rate in band *j*. In order to have a nearly one true positive rate, i.e. $\text{ TPR }\ge 1-\epsilon$ for a small $\epsilon$, we select

13 $$\begin{aligned} J \ge \frac{-\ln \epsilon }{\alpha }, \end{aligned}$$

With (13), the overall expected computational complexity of Algorithm 1 is

14 $$\begin{aligned} O( \log (\epsilon ) ( (M\gamma ^x+N\gamma ^y) + \beta MN ) / \alpha ) \end{aligned}$$

where *M* is the number of sequences in ${\mathcal {X}}$, *N* is the number of sequences in ${\mathcal {Y}}$, $\beta$ is defined as the false positive rate in band *j*, and $\gamma ^x,\gamma ^y$ are defined as expected numbers of buckets that sequences *X* and *Y* in band *j* fall into, respectively.

### Sequence alignment model

Algorithm 1 is limited to standard HMMs, and does not generalize to other latent variable models. Unfortunately, standard HMMs are not sufficient for modelling the important features of the sequence alignment problem, as they can not model insertions and deletions. Here, we define a natural probabilistic model (very similar to pair-HMMs) that we refer to as the sequence alignment model, that takes into account insertions, deletions, and matches/mismatches between pairs of sequences. Our probabilistic model first generates a latent variable *H*, e.g. $H = mmdmmimi$. Here, “*m*”, “*i*”, and “*d*” represent match/mismatch, insertion, and deletion respectively. Given *H*, the generative model generates pre-sequences ${\bar{X}}_{H}$ and ${\bar{Y}}_{H}$, e.g. ${\bar{X}}_{H} = AGCGT-A-$ and ${\bar{Y}}_{H}=AG-TTGAC$. Note that whenever the $t^{th}$ entry of *H* is “*i*” (“*d*”), the corresponding entry ${\bar{X}}_{H,t}$ (resp. ${\bar{Y}}_{H,t}$) is “−” . The model generates *X* and *Y* by removing all “−” from ${\bar{X}}_{H,t}$ and ${\bar{Y}}_{H,t}$ (Fig. 2). In this example, we have:

15 $$\begin{aligned} {\mathbb {P}}(X,Y\mid H)&=P_m(A,A)P_m(G,G)P_d(C)P_m(G,T)\nonumber \\&\quad P_m(T,T)P_i(G)P_m(A,A)P_i(C) \end{aligned}$$

16 $$\begin{aligned} P(H)&=q_{match}q_{match}q_{deletion}q_{match}q_{match}\nonumber \\&\quad q_{insertion}q_{match}q_{insertion} \end{aligned}$$

with $P_{m}:\{A,C,G,T\}\times \{A,C,G,T\}\rightarrow [0,1], P_{i},P_{d}:\{A,C,G,T\}\rightarrow [0,1]$, and $q_{match}$, $q_{insertion}$ and $q_{deletion}$ are positive, $q_{match} + q_{insertion}+ q_{deletion} = 1$ (Fig. 2). Here $P_{m}$, $P_{d}$ and $P_{i}$ are the emission probabilities when the latent variable is match, deletion, and insertion respectively. $q_{match}$, $q_{insertion}$, $q_{deletion}$ are probabilities of the latent variables. Note that $q_{match}$ represents the probability for both match and mismatch.

For simplicity we have assumed that insertion, deletion, and match/mismatch events are happening independent of each other. In practice, these events are usually not independent, and a HMM is used for modeling intervals of insertions and deletions, that is equivalent to using an affine gap penalty. While in this paper we will focus on the independent insertion and deletion model, the algorithms presented can generalize to arbitrary priors.

So far, we have shown that the sequence alignment model can be stated as a special case of latent variable models. We will show how the algorithms we have developed for HMMs (without insertions/deletions) can be adapted to the case of sequence alignment models.

### Efficient sequence alignment by sub-quadratic inference in sequence alignment model

Here we develop analogous methods to solve the inference problem in case of the sequence alignment model. We present a method to align sequences via the bucketing strategy (Algorithm 4), and we detail how to construct optimal buckets to minimize the runtime (Algorithm 5). Our model relies on pair-HMMs, which are different from standard HMMs in that they can also incorporate insertions and deletions. An efficient bucketing strategy is designed using a decision graph structure (Fig. S4). Decision graphs are iteratively grown and pruned, in order to optimize the theoretical complexity (Supplementary Sect. 9.6).

## Supplementary Information

Supplementary Information.

## Data Availability

All data generated or analysed during this study are included in this published article and its supplementary information files.

## Code availability

The command-line version of DSB, available from https://github.com/mohimanilab/DistributionSensitiveBucketing, is capable of conducting searches in both all versus all mode (when searching many reads against each other) and all versus one mode (searching many reads against a single genome).
