# Supplementary material for: Large scale sequence alignment via efficient inference in generative models
Source: Sci Rep. 2023 May 4;13:7285. doi: 10.1038/s41598-023-34257-x (PMC10160065; doi:10.1038/s41598-023-34257-x)
Supplement: Supplementary file 1 — Supplementary Information. [file 41598_2023_34257_MOESM1_ESM.pdf]

Supplementary

S9.1 Proofs for Algorithm 2 True Positive Rates and Complexity

Given a HMM $\mathbb{P}$, a set of buckets $V_{buckets}$ along with corresponding bucketing functions h^x and h^y, let's define α, β, γ^x, and γ^y as :

- $\alpha = P(h^x_j(X) \cap h^y_j(Y) \neq \emptyset \mid (X, Y) \sim \mathbb{P})$ (31)
- $\beta = P(h^x_j(X) \cap h^y_j(Y) \neq \emptyset \mid (X, Y) \sim \mathbb{P}^x\mathbb{P}^y)$ (32)
- $\gamma^x = \mathbb{E}|h^x_j(X)|$ (33)
- $\gamma^y = \mathbb{E}|h^y_j(X)|$ (34)

for $j \in \mathcal{J}$ (we assume that statistics are the same for all j). Moreover, from (8), (9), (10), and (11) (in main text):

- $|h^x_j(X) \cap h^y_j(Y)| \leq 1$ (35)

Intuitively, this means that in each band, each pair of data points cannot both appear in more than one bucket. Using this constraint, we can interpret α, β, γ^x and γ^y as true positive rate, false positive rate, and the expected number of buckets that data points fall into, in each band:

$$\alpha = \sum_{v \in V_{buckets}} P(X \in v, Y \in v \text{ in band } j \mid (X, Y) \sim \mathbb{P}) \tag{36}$$
$$= \text{true positive rate in band } j \tag{37}$$

$$\beta = \sum_{v \in V_{buckets}} P(X \in v, Y \in v \text{ in band } j \mid (X, Y) \sim \mathbb{P}^x\mathbb{P}^y) \tag{38}$$
$$= \text{false positive rate in band } j \tag{39}$$
$$\gamma^x = \mathbb{E}[\# \text{ buckets that } X \text{ falls into, in band } j \mid X \sim \mathbb{P}^x] \tag{40}$$
$$\gamma^y = \mathbb{E}[\# \text{ buckets that } Y \text{ falls into, in band } j \mid Y \sim \mathbb{P}^y] \tag{41}$$

For details of (36) – (41), see Supplementary Note 2. We can now use (36) – (41) to compute the complexity of Algorithm 1. The complexity of Algorithm 1 can be divided into three parts: (i) finding the buckets to which X and Y are mapped, (ii) inserting sequences X and Y into these buckets, and (iii) checking all the pairs (X, Y) that fall into the same buckets. Using prefix trees for mapping, the complexity is bounded by:

$$c_{prefix}(M + N) \cdot \mathit{MaxDepth} \tag{42}$$

where M is the number of sequences in $|\mathcal{X}|$, N is the number of sequences in $|\mathcal{Y}|$, $\mathit{MaxDepth}$ is the maximum depth of the prefix tree, and c_{prefix} is the complexity of progressing one node further in the prefix tree. Inserting sequences into appropriate buckets has expected complexity

$$c_{insert}(M\gamma^x + N\gamma^y) \tag{43}$$

where c_{insert} is the computational cost of inserting a single sequence into a bucket. Therefore, the total expected complexity per band is :

$$c_{prefix}M \cdot MaxDepth + c_{prefix}N \cdot MaxDepth + c_{insert}(M\gamma^x + N\gamma^y) + c_{compute}(\alpha \# TP + \beta \# FP) \tag{44}$$

where $c_{compute}$ denotes complexity of evaluating $\frac{\mathbb{P}(X,Y)}{\mathbb{P}^x(X)\mathbb{P}^y(Y)}$ via the forward algorithm, $\#TP$ is the number of jointly generated pairs and $\#FP$ is the number of independent pairs among all pairs ($\#TP + \#FP = MN$). Here we assume nearly all pairs are false, e.g. $\#TP \ll \#FP$ and $\#FP \approx MN$. Also note that c_{insert}, $c_{compute}$ and c_{prefix} are constants not depending on M or N. In practice, many of the true pairs are missed if we only use a single band, and multiple bands are needed to achieve near one true positive rates. Using J bands, the true positive rate is:

$$TPR = 1 - (1-\alpha)^J \geq 1 - e^{-\alpha J} \tag{45}$$

This is because the chance of a true pair not being captured in each band is $1 - \alpha$, and we further assume the event that true pairs are captured in different bands are independent. In order to have a nearly one true positive rate, i.e. $TPR \geq 1 - \epsilon$ for a small ϵ, using (45) we can select

$$J \geq \frac{-\ln \epsilon}{\alpha}, \tag{46}$$

Thus the overall expected computational complexity of Algorithm 1 is

$$\frac{-\ln \epsilon}{\alpha}\Big(c_{prefix}(M+N) \cdot MaxDepth + c_{insert}(M\gamma^x + N\gamma^y) + \beta MN c_{compute}\Big) \tag{47}$$

where c_{prefix} and c_{insert} are $O(1)$, and $c_{compute}$ is equal to $O(T|\mathcal{H}|^2)$, runtime of the forward algorithm, in case of HMMs. Given that c_{prefix}, c_{insert}, $c_{compute}$ and $MaxDepth$ are constants not growing with M and N, the complexity is:

$$O(\log(\epsilon)((M\gamma^x + N\gamma^y) + \beta MN)/\alpha) \tag{48}$$

In practice, we observe $MaxDepth$ grows logarithmically with the number of data points. Here, we assume $log(M)$ and $log(N)$ are small.

S9.2 Supplementary Note 1. String alignment by Maximum Inner Products Search

Maximum Inner Products Search (MIPS) refers to finding the maximum vector p in a data collection S such that it maximizes the inner product with a query vector q of the same dimension. In the case of DNA sequences, we try to find the sequence X in the sequence collection that maximizes the joint probability of appearing with query sequence Y.

To do this we calculate the joint probability of generating sequence X and sequence Y given a latent variable sequence h, which is a vector consisting of matches, insertions and deletions. This probability can be expressed as the sum over all possible latent variables that could generate the sequence pair (X, Y), and thus can be expressed as an inner product of two vectors (embeddings of X and Y). Hence, in theory it is possible to use MIPS for solving our statistical inference problem.

However, the limitation is that we one needs to convert any string to its vector embedding. Since the latent variable sequences that could generate a sequence have 3^n cases for a string of length n (corresponding to match, insertion, and deletion), the time and space required to perform the conversion also grow exponentially. Therefore, in practice MIPS is computationally prohibitive for solving the string alignment problem for strings with length above ~ 20.

S9.3 Supplementary Note 2. True Positive and True Negative Rates

The true positive rate of Algorithm 2 in a single band is:

$$
\begin{aligned}
\alpha &= P(h^x(X) \cap h^y(Y) \neq \emptyset \mid (X,Y) \sim \mathbb{P}) && (49)\\
&= \sum_{i=1}^{D} P(i \in h^x(X),\ i \in h^y(Y) \mid (X,Y) \sim \mathbb{P}) && (50)\\
&= \sum_{i=1}^{D} P(X \in v_i, Y \in v_i \mid (X,Y) \sim \mathbb{P}) && (51)\\
&= \text{fraction of true pairs being called positive} && (52)\\
&= \text{true positive rate} && (53)
\end{aligned}
$$

where (50) holds because of (35). Similarly the false positive rate in a single band is:

$$
\begin{aligned}
\beta &= P(h^x(X) \cap h^y(Y) \neq \emptyset \mid (X,Y) \sim \mathbb{P}^x\mathbb{P}^y) && (54)\\
&= \sum_{i=1}^{D} P(i \in h^x X,\ i \in h^y(Y) \mid (X,Y) \sim \mathbb{P}^x\mathbb{P}^y) && (55)\\
&= \sum_{i=1}^{D} P(X \in v_i, Y \in v_i \mid (X,Y) \sim \mathbb{P}^x\mathbb{P}^y) && (56)\\
&= \text{fraction of random pairs being called positive} && (57)\\
&= \text{false positive rate} && (58)
\end{aligned}
$$

where once again (55) holds because of (35). Moreover, note that

$$
\begin{aligned}
\gamma^x &= \mathbb{E}|h^x(X)| = \sum_{i=1}^{D} P(i \in h^x(X) \mid X \sim \mathbb{P}^x) && (59)\\
&= \sum_{i=1}^{D} P(X \in v_i \mid X \sim \mathbb{P}^x) && (60)\\
&= \mathbb{E}[\text{number of buckets that } X \text{ falls into} \mid X \sim \mathbb{P}^x] && (61)
\end{aligned}
$$

and similarly

$$
\gamma^y = \mathbb{E}[\text{number of buckets that } X \text{ falls into} \mid Y \sim \mathbb{P}^y] \quad (62)
$$

S9.4 Supplementary Note 3. Dynamic Programming For Sequence Alignment Model

$$\begin{aligned}
&\Phi(S_1, S_2) = \mathbb{P}(S_1 \text{ Prefix of X, } S_2 \text{ Prefix of Y}) \\
&= \sum_{T_3=1}^{T_1+T_2} \mathbb{P}(S_1 \text{ Prefix of X, } S_2 \text{ Prefix of Y} \mid h_{T_3} = \text{match}) \\
&\qquad \mathbb{P}(h_{T_3} = \text{match})\mathbb{P}(T_3) \\
&+ \sum_{T_3=1}^{T_1+T_2} \mathbb{P}(S_1 \text{ Prefix of X, } S_2 \text{ Prefix of Y} \mid h_{T_3} = \text{insertion}) \\
&\qquad \mathbb{P}(h_{T_3} = \text{insertion})\mathbb{P}(T_3) \\
&+ \sum_{T_3=1}^{T_1+T_2} \mathbb{P}(S_1 \text{ Prefix of X, } S_2 \text{ Prefix of Y} \mid h_{T_3} = \text{deletion}) \\
&\qquad \mathbb{P}(h_{T_3} = \text{deletion})\mathbb{P}(T_3)
\end{aligned} \tag{63}$$

$$\begin{aligned}
&= \sum_{T_3=1}^{T_1+T_2} \mathbb{P}(S_1[1:T_1-1] \text{ Prefix of X, } S_2[1:T_2-1] \text{ Prefix of Y}) \\
&\qquad P_m(S_1(T_1), S_2(T_2))\mathbb{P}(h_{T_3} = \text{match})\mathbb{P}(T_3) \\
&+ \sum_{T_3=1}^{T_1+T_2} \mathbb{P}(S_1[1:T_1] \text{ Prefix of X, } S_2[1:T_2-1] \text{ Prefix of Y}) \\
&\qquad P_i(S_2(T_2))\mathbb{P}(h_{T_3} = \text{insertion})\mathbb{P}(T_3) \\
&+ \sum_{T_3=1}^{T_1+T_2} \mathbb{P}(S_1[1:T_1-1] \text{ Prefix of X, } S_2[1:T_2] \text{ Prefix of Y}) \\
&\qquad P_d(S_1(T_1))\mathbb{P}(h_{T_3} = \text{deletion})\mathbb{P}(T_3)
\end{aligned} \tag{64}$$

$$\begin{aligned}
&= \sum_{T_3=1}^{T_1+T_2} \Phi(S_1[1:T_1-1], S_2[1:T_2-1])P_m(S_1(T_1), S_2(T_2)) \\
&\qquad \mathbb{P}(h_{T_3} = \text{match})\mathbb{P}(T_3) \\
&+ \sum_{T_3=1}^{T_1+T_2} \Phi(S_1[1:T_1], S_2[1:T_2-1])P_i(S_2(T_2)) \\
&\qquad \mathbb{P}(h_{T_3} = \text{insertion})\mathbb{P}(T_3) \\
&+ \sum_{T_3=1}^{T_1+T_2} \Phi(S_1[1:T_1-1], S_2[1:T_2])P_d(S_1(T_1)) \\
&\qquad \mathbb{P}(h_{T_3} = \text{deletion})\mathbb{P}(T_3)
\end{aligned} \tag{65}$$

$$\begin{aligned}
&= \Phi(S_1[1:T_1-1], S_2[1:T_2-1])q_{match}P_m(S_1(T_1), S_2(T_2)) \\
&+\Phi(S_1[1:T_1], S_2[1:T_2-1])q_{insertion}P_i(S_2(T_2)) \\
&+\Phi(S_1[1:T_1-1], S_2[1:T_2])q_{deletion}P_d(S_1(T_1))
\end{aligned} \tag{66}$$

Similarly, we have

$$
\begin{aligned}
&\Psi^x(S_1) = \mathbb{P}(S_1 \text{ Prefix of X}) \\
&= \sum_{T_3=1}^{T_1+T_2} \mathbb{P}(S_1 \text{ Prefix of X } \mid h_{T_3} = \text{match}) q_{match} P(T_3) \\
&+ \sum_{T_3=1}^{T_1+T_2} \mathbb{P}(S_1 \text{ Prefix of X } \mid h_{T_3} = \text{insertion}) q_{insertion} P(T_3) \\
&+ \sum_{T_3=1}^{T_1+T_2} \mathbb{P}(S_1 \text{ Prefix of X } \mid h_{T_3} = \text{deletion}) q_{deletion} P(T_3) \qquad (67) \\
&= \sum_{T_3=1}^{T_1+T_2} \mathbb{P}(S_1[1:T_1-1] \text{ Prefix of X}) q_{match} P_m^x(S_1(T_1)) P(T_3) \\
&+ \sum_{T_3=1}^{T_1+T_2} \mathbb{P}(S_1[1:T_1] \text{ Prefix of X}) q_{insertion} P(T_3) \\
&+ \sum_{T_3=1}^{T_1+T_2} \mathbb{P}(S_1[1:T_1-1] \text{ Prefix of X}) q_{deletion} P_d(S_1(T_1)) P(T_3) \qquad (68) \\
&= \Psi^x(S_1[1:T_1-1]) q_{match} P_m^x(S_1(T_1)) \\
&+\Psi^x(S_1[1:T_1]) q_{insertion} \\
&+\Psi^x(S_1[1:T_1-1]) q_{deletion} P_d(S_1(T_1)) \qquad (69)
\end{aligned}
$$

Therefore we have

$$
\begin{aligned}
\Psi^x(S_1) = &\frac{q_{match} P_m^x(S_1(T_1)) + q_{deletion} P_d(S_1(T_1))}{1 - q_{insertion}} \\
&\Psi^x(S_1[1:T_1-1]) \qquad (70)
\end{aligned}
$$

Similarly

$$
\begin{aligned}
\Psi^y(S_2) = &\frac{q_{match} P_m^y(S_2(T_2)) + q_{insertion} P_i(S_2(T_2))}{1 - q_{deletion}} \\
&\Psi^y(S_2[1:T_2-1]) \qquad (71)
\end{aligned}
$$

Here, P_m^x and P_m^y denote marginals of P_m. Note that in the special case where $P_m^x = P_d$ and $P_m^y = P_i$

$$
\Psi^x(S_1) = \prod_{t_1=1}^{T_1} P_m^x(S_1(t_1)) \qquad (72)
$$

and

$$
\Psi^y(S_2) = \prod_{t_2=1}^{T_2} P_m^y(S_2(t_2)) \qquad (73)
$$

S9.5 Supplementary Note 4. PBSIM Simulation Details

We use PBSIM to generate reads for Experiment 3 with the following specifications:

- data-type: CLR
- depth: 10
- length-mean: 700
- length-sd: 150
- length-min: 400
- length-max: 1000
- accuracy-mean: $[ACC]$
- accuracy-sd: 0.07
- accuracy-min: $[ACC] - 0.1$
- accuracy-max: $[ACC] + 0.1$
- difference-ratio: 10:60:30

The depth denotes the depth of coverage. $[ACC]$ denotes the mean accuracy for a condition. We examine conditions where $[ACC]$ ranges from 0.55 to 0.75 (equivalent to error rates from 0.25 to 0.45). The difference ratio denotes the distribution of substitution/insertion/deletion in the simulation errors. The value used is the default for CLR data-type.

S9.6 Technical details for Efficient Sequence Alignment by Sub-quadratic Inference in Sequence Alignment Model.

In the sequence alignment model, $X \in \mathcal{A}^{T_1}$ and $Y \in \mathcal{B}^{T_2}$ are dependent on a latent variable $H \in \mathcal{H}^{T_3}$ where $\mathcal{H} = \{m, i, d\}$ and $\max(T_1, T_2) \leq T_3 \leq T_1 + T_2$ as follows:

$$\mathbb{P}(X, Y) = \sum_{T_3} \sum_{H \in \mathcal{H}^{T_3}} \mathbb{P}(X, Y \mid H) P(H) \tag{74}$$

where

$$\mathbb{P}(X, Y \mid H) = \mathbb{P}(\bar{X}_H, \bar{Y}_H \mid H) = \prod_{t=1}^{T_3} P(\bar{X}_{H,t}, \bar{Y}_{H,t} \mid h_t) \tag{75}$$

$$\mathbb{P}(H) = \prod_{T=1}^{T_3} P(h_t) \tag{76}$$

In (75), $\bar{X}_H \in \{\mathcal{A} \cup \{-\}\}^{T_3}$ and $\bar{Y}_H \in \{\mathcal{B} \cup \{-\}\}^{T_3}$, h_t is the tth entry in $\mathcal{H}$, and $\bar{X}_{H,t}$ is the tth entry in $\bar{X}_H$, and:

$$\bar{X}_{H,t} = \begin{cases} x_{t-i(t)}, & \text{if } h_t \neq \text{“}i\text{”} \\ -, & \text{if } h_t = \text{“}i\text{”} \end{cases}$$

$$\bar{Y}_{H,t} = \begin{cases} y_{t-d(t)}, & \text{if } h_t \neq \text{“}d\text{”} \\ -, & \text{if } h_t = \text{“}d\text{”} \end{cases}$$

where $i(t)$ (resp. $d(t)$) is the number of insertions (resp. deletions) before s. Moreover, $P(h_t = \text{“}m\text{”}) + P(h_t = \text{“}i\text{”}) + P(h_t = \text{“}d\text{”}) = 1$.

We use dynamic programming to compute $\Phi(S_1, S_2)$ for each string $S_1 \in \mathcal{A}^{T_1}$ and $S_2 \in \mathcal{A}^{T_2}$. For any string S, let $S[1:t]$ denote the prefix substring of S of length t. It can be

shown that

$$\begin{aligned}\Phi(S_1,S_2) = \Phi(S_1([1:T_1-1],S_2[1:T_2])q_{deletion}P_d(S_1(T_1))+\\ \Phi(S_1([1:T_1],S_2[1:T_2-1])q_{insertion}P_i(S_2(T_2))+\\ \Phi(S_1([1:T_1-1],S_2[1:T_2-1])q_{match}P_m(S_1(T_1),S_2(T_2))\end{aligned} \tag{77}$$

(See Supplementary Note 3 for proof). For instance:

$$\begin{aligned}\Phi(AA,CA) =& q_{deletion}\Phi(A,CA)P_d(A) + q_{match}\Phi(A,C)\\ & P_m(A,A) + q_{insertion}\Phi(AA,C)P_i(A)\end{aligned} \tag{78}$$

Similarly, Ψ^x and Ψ^y can be computed recursively (See Supplementary Note 3). Note that in (77), the joint probability Φ at each node depend on three other nodes (referred to as parents). Thus, as opposed to the decision trees in case of HMMs, our data structure is a decision graph (i.e. a directed acyclic graph). Moreover, in contrast to the case of HMMs where the label of edges were limited to $|\mathcal{A}| \times |\mathcal{B}|$, here the label of edges are from $\mathcal{A} \times \mathcal{B} \cup \mathcal{A} \times \{-\} \cup \{-\} \times \mathcal{B}$ (Figure S4).

Now, similar to the case of standard HMMs, we construct $V_{buckets}$ via a directed graph where we accept a node w if $\frac{\Phi(w.S_x,w.S_u)}{\Psi^x(w.S_x)\Psi^y(w.S_y)}$ is high, and prune it if $\frac{\Phi(w.S_x,w.S_u)}{\Psi^x(w.S_x)}$ or $\frac{\Phi(w.S_x,w.S_u)}{\Psi^y(w.S_y)}$ is low.

Algorithm 3 describes a strategy for sub-quadratic sequence alignment given a decision graph and a set of buckets (analogous to Algorithm 1). Algorithm 5 describes how to design the decision graph and the set of buckets to minimize the complexity (analogous to Algorithm 2).

As using a single band usually results in low true positive rates, similar to the case of HMMs we use multiple bands (Figure S5). However, Algorithm 3 differs from Algorithm 1 in that we call the pair (X, Y) a positive if both sequences fall into the same bucket in any pair of bands. In contrast, in Algorithm 1, (X, Y) is called a positive whenever X and Y fall into the same bucket in the same band. We use this strategy because in contrast to the HMM problem, in the sequence alignment problem we have data points that are not aligned (i.e. they could have different lengths).

Selecting Decision Trees.

A naive choice of the decision tree and buckets could lead to high false negative rates or inefficient runtime of Algorithm 1. Here, we propose an algorithm for constructing the decision tree and the buckets utilized in Algorithm 1 to minimize the complexity in (14) while maintaining a near perfect true positive rate. It is clear from (14) that in order to keep the complexity of the tree low, $V_{buckets}$ should be designed in a way that

$$\max\left\{MN\frac{\beta}{\alpha}, N\frac{\gamma^x}{\alpha}, M\frac{\gamma^y}{\alpha}\right\} \tag{79}$$

is small. If we define $\Phi(S_1, S_2)$, $\Psi^x(S_1)$ and $\Psi^y(S_2)$ as:

$$\begin{aligned}\Phi(\mathrm{S}_1,\mathrm{S}_2) =& P(\mathrm{S}_1 \text{ is a prefix of } X\\ & \text{and } \mathrm{S}_2 \text{ is a prefix of } Y) \qquad (80)\\ \Psi^x(\mathrm{S}_1) =& P(\mathrm{S}_1 \text{ is a prefix of } X) \qquad (81)\\ \Psi^y(\mathrm{S}_2) =& P(\mathrm{S}_2 \text{ is a prefix of } Y) \qquad (82)\end{aligned}$$

Then from (36)-(41) (see supplementary materials) we have:

$$\alpha = \sum_{v \in V_{buckets}} \Phi(v.S_x, v.S_y) \tag{83}$$

$$\gamma^x = \sum_{v \in V_{buckets}} \Psi^x(v.S_x) \tag{84}$$

$$\gamma^y = \sum_{v \in V_{buckets}} \Psi^y(v.S_y) \tag{85}$$

$$\beta = \sum_{v \in V_{buckets}} \Psi^x(v.S_x)\Psi^y(v.S_y) \tag{86}$$

Thus, the decision of whether to prune a node, add it to $V_{buckets}$, or branch it to more children is done in the following way (i.e., Algorithm 2):

1. designate a node v as a bucket if $\frac{\Phi(v.S_x,v.S_y)}{\Psi^x(v.S_x)\Psi^y(v.S_y)} \geq c_0 NM$.

2. prune a node if $\frac{\Phi(v.S_x,v.S_y)}{\Psi^x(v.S_x)} \leq c_x N$ or $\frac{\Phi(v.S_x,v.S_y)}{\Psi^y(v.S_y)} \leq c_y M$.

3. otherwise (if none of the above holds), branch the node to $|\mathcal{A}| \times |\mathcal{B}|$ children.

where c_0, c_x and c_y are constants. In HMMs, $\Phi(v.S_x, v.S_y)$, $\Psi^x(v.S_x)$ and $\Psi^y(v.S_y)$ can be computed via dynamic programming.

S9.7 Supplementary Algorithms

Algorithm 1 *Efficient database search for HMMs.*

Input: Alphabets $\mathcal{A}$, $\mathcal{B}$, HMM $\mathbb{P}(x, y)$, set of buckets $V_{buckets}$, bands $\mathcal{J} \subseteq \{1 \ldots J\}$, $\mathcal{X} = \{X^1, ..., X^N\} \subseteq \mathcal{A}^T$, $\mathcal{Y} = \{Y^1, ..., Y^M\} \in \mathcal{B}^T$, and threshold $\Delta \in \mathbb{R}^+$.
Output: All pairs of sequences $X, Y \in \mathcal{X} \times \mathcal{Y}$ satisfying $\frac{\mathbb{P}(X,Y)}{\mathbb{P}^x(X)\mathbb{P}^y(Y)} > \Delta$.
Preprocessing: Construct two prefix trees for $V_{buckets}$ based on S_x and S_y:
For $j \in \mathcal{J}$:
    $map(\mathcal{X}, \mathcal{Y}, j)$
Procedure $map(\mathcal{X}, \mathcal{Y}, j)$:
    For X in $\mathcal{X}$:
        For $\{v \in V_{buckets} \mid \text{Prefix}^x(v, X, j) = 1\}$:
            $v.insert_x(X)$.
    For Y in $\mathcal{Y}$:
        For $\{v \in V_{buckets} \mid \text{Prefix}^y(v, Y, j) = 1\}$:
            $v.insert_y(Y)$.
    For $v \in V_{buckets}$:
        For $X \in v$:
            For $Y \in v$:
                Compute $\frac{\mathbb{P}(X,Y)}{\mathbb{P}^x(X)\mathbb{P}^y(Y)}$ by forward algorithm presented in ALGORITHM 4.
                Call (X, Y) a positive and report a pair if $\frac{\mathbb{P}(X,Y)}{\mathbb{P}^x(X)\mathbb{P}^y(Y)} > \Delta$.

Algorithm 2 *Constructing $V_{buckets}$ for HMMs.*

Input: bucketing and termination thresholds c_0, c_x and c_y.
Output: A decision tree $G = (V, E)$, and buckets $V_{buckets}$.
Step 1: Initialize *root* node.
  Define $root.S_x = root.S_y = \emptyset$.
  Define $\Phi(\emptyset, \emptyset, h) = \Psi^x(\emptyset, h) = \Psi^y(\emptyset, h) = 1/|\mathcal{H}|$ for $h \in \mathcal{H}$.
Step 2: ConstructTree($root$)
Procedure: ConstructTree(v)
  For $a \in \mathcal{A}$:
    For $b \in \mathcal{B}$:
      Create a new node w
      Set w as the child of v through edge (a, b)
      $w.S_x = v.s_x + a$
      $w.S_y = v.s_y + b$
      For $h \in \mathcal{H}$:
        $\Phi(w.S_x, w.S_y, h) \leftarrow \sum_{h' \in \mathcal{H}} \Phi(v.S_x, v.S_y, h') P_{trans}(h \mid h') P_{emit}(a, b \mid h)$
        $\Psi^x(w.S_x, h) \leftarrow \sum_{h' \in \mathcal{H}} \Psi^x(v.S_x, h') P_{trans}(h \mid h') P^x_{emit}(a \mid h)$
        $\Psi^y(w.S_y, h) \leftarrow \sum_{h' \in \mathcal{H}} \Psi^y(v.S_y, h') P_{trans}(h \mid h') P^y_{emit}(b \mid h)$
      $\Phi(w.S_x, w.S_y) \leftarrow \sum_{h \in \mathcal{H}} \Phi(w.S_x, w.S_y, h)$
      $\Psi^x(w.S_x) \leftarrow \sum_{h \in \mathcal{H}} \Psi^x(w.S_x, h)$
      $\Psi^y(w.S_y) \leftarrow \sum_{h \in \mathcal{H}} \Psi^y(w.S_y, h)$
      If $(\frac{\Phi(w.S_x, w.S_y)}{\Psi^x(w.S_x)\Psi^y(w.S_y)} > c_0 NM)$:
        then $V_{buckets}.insert(w)$ # accept bucket
      Else If $(\frac{\Phi(w.S_x, w.S_y)}{\Psi^y(w.S_y)} < c_y M)$
        then prune w #do nothing
      Else If $(\frac{\Phi(w.S_x, w.S_y)}{\Psi^x(w.S_x)} < c_x N)$:
        then prune w #do nothing
      Else then ConstructTree(w) # spawn

Algorithm 3 *Sequence alignment via bucketing*

Input: The latent variable model $\mathbb{P}$, threshold Δ, buckets $V_{buckets}$, bands $\mathcal{J} = \{1, \cdots, J\}$, $\mathcal{X} = \{X^1, ..., X^N\} \subseteq \mathcal{A}^S$ and $\mathcal{Y} = \{Y^1, ..., Y^M\} \in \mathcal{B}^S$.
Output: All pairs of sequences $X, Y \in \mathcal{X} \times \mathcal{Y}$ satisfying $\frac{\mathbb{P}(X,Y)}{\mathbb{P}^x(X)\mathbb{P}^y(Y)} > \Delta$.
For $j \in \mathcal{J}$:
  For X in $\mathcal{X}$:
    For $\{v \in V_{buckets} \mid \text{Prefix}^x(v, X, j) = 1\}$:
      $v.insert_x(X)$.
  For Y in $\mathcal{Y}$:
    For $\{v \in V_{buckets} \mid \text{Prefix}^y(v, Y, j) = 1\}$:
      $v.insert_y(Y)$.
For $v \in V_{buckets}$:
  For $X \in v$:
    For $Y \in v$:
      Call (X, Y) a positive
      Report the pair if $\frac{\mathbb{P}(X,Y)}{\mathbb{P}^x(X)\mathbb{P}^y(Y)} > \Delta$.

Algorithm 4 *Brute force solution to inference problem in case of HMMs using forward algorithm.*

Input: A threshold Δ, database $\mathcal{X} = \{X^1, \ldots, X^N\}$ and queries $\mathcal{Y} = \{Y^1, \ldots, Y^M\}$.
Output: All pairs $(X, Y) \in \mathcal{X} \times \mathcal{Y}$ that are likely to be produced by the HMM.
Step 1: **For** $X \in \mathcal{X}$:
  For $Y \in \mathcal{Y}$:
    Run ForwardAlgorithm(X, Y) to compute
    $\mathbb{P}(X, Y)$, $\mathbb{P}^x(X)$, and $\mathbb{P}^y(Y)$.
    Report (X, Y) if $\frac{\mathbb{P}(X,Y)}{\mathbb{P}^x(X)\mathbb{P}^y(Y)} > \Delta$.

Procedure: ForwardAlgorithm(X, Y)
  Step 1: Initialize $\rho(X, Y, 0, h) \leftarrow 1/|\mathcal{H}|$,
    $\rho^x(X, 0, h) \leftarrow 1/|\mathcal{H}|$, $\rho^y(Y, 0, h) \leftarrow 1/|\mathcal{H}|$
    for all h.
  Step 2: **For** $t \in \{1, ..., T\}$:
    For $h \in \mathcal{H}$:
      $\rho(X, Y, t, h) \leftarrow 0$
      $\rho^x(X, t, h) \leftarrow 0$
      $\rho^y(Y, t, h) \leftarrow 0$
      For $h' \in \mathcal{H}$:
        $\rho(X, Y, t, h) \leftarrow \rho(X, Y, t, h)$
        $+\rho(X, Y, t-1, h') \cdot P_{trans}(h \mid h')\cdot$
        $P_{emit}(x_t, y_t \mid h)$

        $\rho^x(X, t, h) \leftarrow \rho^x(X, t, h)$
        $+\rho^x(X, t-1, h') \cdot P_{trans}(h \mid h')\cdot$
        $P^x_{emit}(x_t \mid h)$

        $\rho^y(Y, t, h) \leftarrow \rho^y(Y, t, h)$
        $+\rho^y(Y, t-1, h') \cdot P_{trans}(h \mid h')\cdot$
        $P^y_{emit}(y_t \mid h)$
  Step 3: $\mathbb{P}(X, Y) \leftarrow \sum_{h \in \mathcal{H}} \rho(X, Y, T, h)$
    $\mathbb{P}^x(X) \leftarrow \sum_{h \in \mathcal{H}} \rho^x(X, T, h)$
    $\mathbb{P}^y(Y) \leftarrow \sum_{h \in \mathcal{H}} \rho^y(Y, T, h)$

Algorithm 5 *Constructing $V_{buckets}$ for sequence alignment model.*

Input: bucketing and termination thresholds c_0, c_x, c_y.
Output: A decision graph, along with a subset of leaf nodes $V_{buckets}$.
Step 1: Initialize root node, $root.S_x = root.S_y = \emptyset$. Define $\Phi(\emptyset, \emptyset) = \Psi^x(\emptyset) = \Psi^y(\emptyset) = 1$
Step 2: ConstructGraph(root)
Procedure ConstructGraph(v)
  For $a \in \mathcal{A}$:
    For $b \in \mathcal{B}$:
      If child node corresponding to (a, b) does not already exist, create node w with $w.S_x = v.S_x + a$, $w.S_y = v.S_y + b$, $\Phi(w.S_x, w.S_y) = 0$
      Set w as child of v through edge (a, b)
      $\Phi(w.S_x, w.S_y) = \Phi(w.S_x, w.S_y)+ \Phi(v.S_x, v.S_y) q_{match} P_m(a, b)$
      $\Psi^x(w.S_x) = \Psi^x(v.S_x) P_m(a)$
      $\Psi^y(w.S_y) = \Psi^y(v.S_y) P_m(b)$
  For a in $\mathcal{A}$:
    If child node corresponding to $(a, -)$ does not exist, create node w with $w.S_x = v.S_x + a$, $w.S_y = v.S_y$ and $\Phi(w.S_x, w.S_y) = 0$
    Set w as child of v through edge $(a, -)$
    $\Phi(w.S_x, w.S_y) = \Phi(w.S_x, w.S_y)+ \Phi(v.S_x, v.S_y) P_{insert} P_i(a)$
    $\Psi^x(w.S_x) = \Psi^x(v.S_x) P^x_m(a)$
  For b in $\mathcal{B}$:
    If child node corresponding to $(-, b)$ does not exist, create node w with $w.S_x = v.S_x$, $w.S_y = v.S_y + b$ and $\Phi(w.S_x, w.S_y) = 0$
    Set w as child of v through edge $(-, b)$
    $\Phi(w.S_x, w.S_y) = \Phi(w.S_x, w.S_y)+ \Phi(v.S_x, v.S_y) q_{deletion} P_d(b)$
    $\Psi^y(w.S_y) = \Psi^y(v.S_y) P^y_m(b)$
  If $\left(\frac{\Phi(w.S_x, w.S_y)}{\Psi^x(w.S_x)\Psi^y(w.S_y)} > c_0 NM\right)$:
    then $V_{buckets}.insert(w)$ #accept bucket
  Else If $\left(\frac{\Phi(w.S_x, w.S_y)}{\Psi^x(w.S_x)} < c_x N \text{ or } \frac{\Phi(w.S_x, w.S_y)}{\Psi^y(w.S_y)} < c_y M\right)$:
    then prune w #do nothing
  Else:
    then ConstructGraph(w) #spawn

S9.8 Additional Tables

E. coli reads against E. coli genome		
	False Negatives	True Positive Rate
DSB-SA	2912	0.613
Minimap2	4232	0.439
DALIGNER	3534	0.531
BlasR	3479	0.538
MMSeqs2	2695	0.642
GraphMap	4435	0.412
Winnowmap	4688	0.378

Table S1 *False Negatives/True Positive Rate analysis of various methods for mapping PacBio long reads of E. coli to the E. coli genome.*

E. coli reads against Citrobacter genome		
	False Negatives	True Positive Rate
DSB-SA	2717	0.534
Minimap2	4150	0.289
DALIGNER	3680	0.369
BlasR	4506	0.228
MMSeqs2	3595	0.383
GraphMap	3811	0.346
Winnowmap	5232	0.103

Table S2 *False Negatives/True Positive Rate analysis of various methods for mapping PacBio long reads of E. coli to the Citrobacter genome.*

E. coli reads against G. Endobia genome		
	False Negatives	True Positive Rate
DSB-SA	726	0.196
Minimap2	848	0.0609
DALIGNER	843	0.0664
BlasR	823	0.089
MMSeqs2	743	0.177
GraphMap	753	0.166
Winnowmap	874	0.032

Table S3 *False Negatives/True Positive Rate analysis of various methods for mapping PacBio long reads of E. coli to the G. Endobia genome.*

S. cerevisiae reads against S. cerevisiae genome		
	False Negatives	True Positive Rate
DSB-SA	55 338	0.560
Minimap2	117 281	0.068
DALIGNER	90 055	0.285
BlasR	111 821	0.111
MMSeqs2	49 940	0.603
GraphMap	119 364	0.052
Winnowmap	118 918	0.0555

Table S4 *False Negatives/True Positive Rate analysis of various methods for mapping PacBio long reads of S. Cerevisiae to the S. Cerevisiae genome.*

H. Sapien Chromosome 12 reads against H. Sapien Chromosome 12 genome		
	False Negatives	True Positive Rate
DSB-SA	18 904	0.549
Minimap2	38 439	0.0422
DALIGNER	31 018	0.227
BlasR	39 489	0.0161
MMSeqs2	19 161	0.522
GraphMap	40 091	0.0011
Winnowmap	39 457	0.016 91

Table S5 *False Negatives/True Positive Rate analysis of various methods for mapping PacBio long reads of H. Sapien Chromosome 12 to the H. Sapien Chromosome 12 genome.*

M. Musculus Chromosome 12 reads against M. Musculus Chromosome 12 genome		
	False Negatives	True Positive Rate
DSB-SA	94	0.824
Minimap2	365	0.3177
DALIGNER	69	0.871
BlasR	326	0.391
MMSeqs2	93	0.826
GraphMap	425	0.206
Winnowmap	399	0.254

Table S6 *False Negatives/True Positive Rate analysis of various methods for mapping PacBio long reads of M. Musculus Chromosome 12 to M. Musculus 12 genome.*

S9.9 Additional Figures

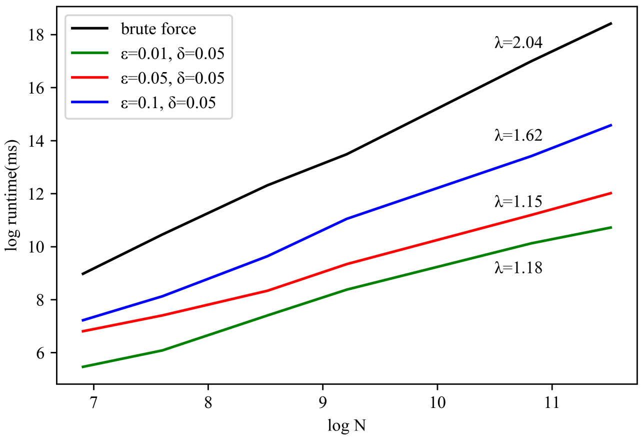

Figure S1 *Log of runtime in milliseconds of DSB-HMM and brute force versus log of number of sequences N for various values of ϵ and $\delta = 0.05$. The parameters are selected in a way that TP $\geq 99\%$ is maintained.*

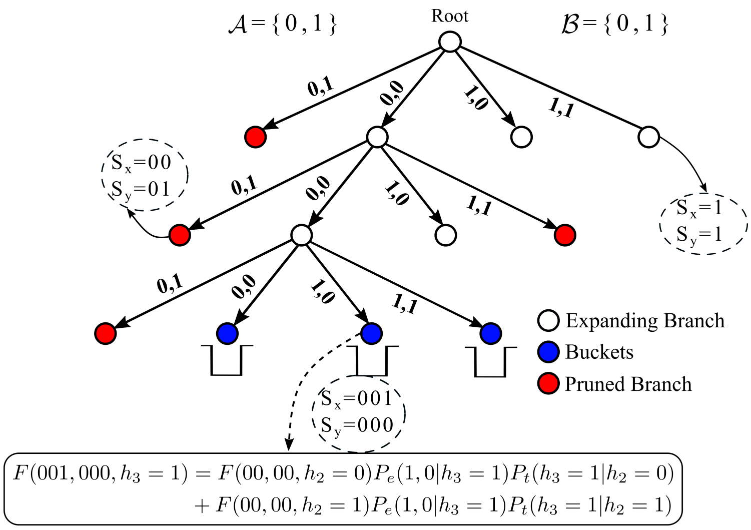

■ ***Figure S2*** *An illustration of HMM decision tree construction. Examples of accepted, pruned, and branched nodes are shown. Refer to Algorithm 3 for further details of the tree construction.*

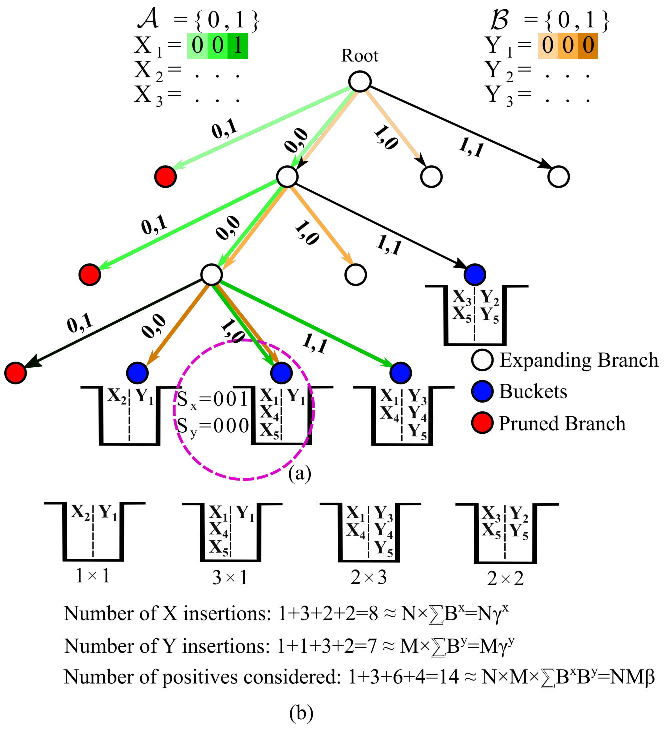

■ ***Figure S3*** *The figure illustrates mapping sequences to buckets through decision trees, and checking collisions between sequences in each bucket (Algorithm 2).* ***(a)*** *The path for sequences $X_1 = 001$ and $Y_1 = 000$ are shown in green and orange, respectively.* ***(b)*** *The complexity of the algorithm involves insertion of sequences $X \in \mathcal{X}$ (here 8 insertions), insertions of sequences $Y \in \mathcal{Y}$ (here 7 insertions) and checking each pair X and Y in each bucket (here 14 checks). Refer to Algorithm 2 for further details.*

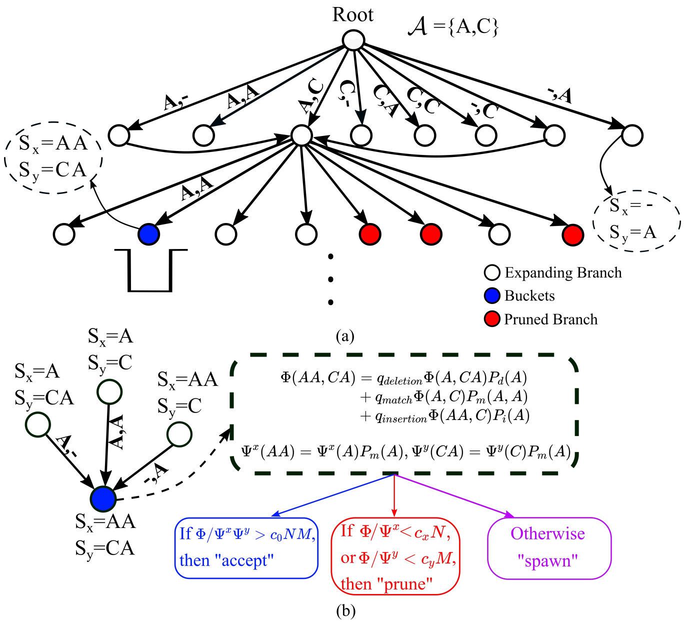

Figure S4 *Graph construction for the string alignment problem with alphabet $\mathcal{A} = \{A, C\}$ (we exclude G and T from the alphabet for simplicity). Each node is either a leaf node, or has eight children, corresponding to $\{(A, A), (A, C), (C, A), (C, C), (A, -), (C, -), (-, A), (-, C)\}$. Because of insertions and deletions, each node of this graph can have up to three parents, e.g. node v corresponding to (AA,CA) has parents (A,C), (AA,C), (A,CA). We compute Φ ,Ψ^x and Ψ^y on the nodes of the graph recursively, starting from the root. If $\frac{\Phi}{\Psi^x\Psi^y}$ exceed a threshold, the corresponding node gets accepted as a bucket, while if $\frac{\Phi}{\Psi^x}$ or $\frac{\Phi}{\Psi^y}$ goes below a threshold, the corresponding node gets pruned. If none of these scenarios holds, the corresponding node gets spawned. For further details, refer to Algorithm 5.*

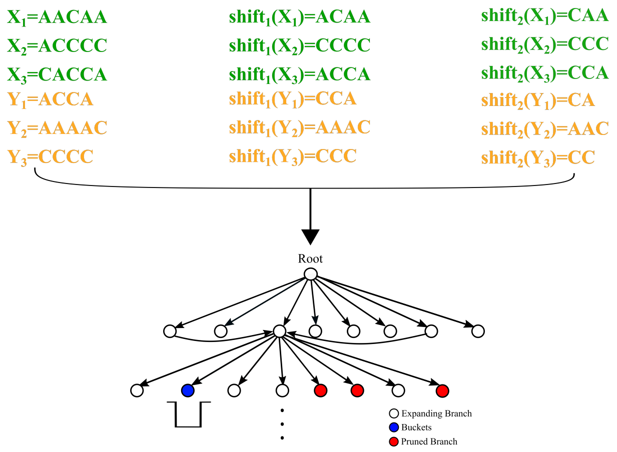

Figure S5 *Running Algorithm 2 on a single band usually results in a large number of false negatives. To alleviate this, we use multiple bands, where in each band we shift sequences. We insert the shifted data into the decision tree, and check all collisions in each band. Refer to Algorithm 4 for further details.*
